# *Strongyloides stercoralis* hyperinfection after corticosteroid therapy: a report of two cases

**DOI:** 10.4103/0256-4947.55172

**Published:** 2009

**Authors:** Mona A. Al Maslamani, Hussam A. Al Soub, Abdel Latif M. Al Khal, Issam A. Al Bozom, Mohammed J. Abu Khattab, Kadavil C. Chacko

**Affiliations:** a From the Department of Medicine, Hamad Medical Corporation, Doha, Qatar; b From the Department of Laboratory Medicine and Pathology, Hamad Medical Corporation, Doha, Qatar

## Abstract

Two cases of *Strongyloides stercoralis* hyperinfection are described. Both patients were expatriates from the Indian subcontinent, and reported the use of corticosteroids. The first patient presented with severe pulmonary disease that necessitated respiratory support, followed by acute abdomen and intestinal obstruction and he succumbed to these diseases. The second patient also presented with acute pulmonary disease, which responded to antihelmintic treatment and supportive care; however, he died later due to his primary disease. The clinical features of *S stercoralis* hyperinfection are nonspecific; therefore, a high index of suspicion is required for early diagnosis and to start appropriate therapy. Because of the seriousness of the disease and the associated high mortality we suggest screening for *S stercoralis* in patients from endemic areas who will be taking immunosuppressive therapy.

*Strongyloides stercoralis* is a ubiquitous soil transmitted intestinal nematode of humans that is endemic in many areas throughout the tropical and temperate regions.^1^,^2^ The term hyperinfection is used to denote autoinfection, a phenomenon in which the number of worms increases tremendously. Development or exacerbation of gastrointestinal and pulmonary symptoms is seen, and the detection of increased numbers of larvae in stool and or sputum is the hallmark of hyperinfection. *S stercoralis* hyperinfection syndrome, well recognized since 1966, has increased in frequency during the past decades as a result of immunosuppressive therapy used in the treatment of organ transplant recipients, collagen vascular diseases and cancer.^3^ Of all the immunosuppressive drugs, corticosteroids are the most widely used and the most specifically associated with transforming chronic strongyloidiasis to hyperinfection.^4^ The outcome in patients with hyperinfection remains poor with 50% mortality despite therapy.^5^ We report two cases of *S stercoralis* hyperinfection following corticosteroid therapy with fatal outcome.

## CASE 1

**Figure 1:**
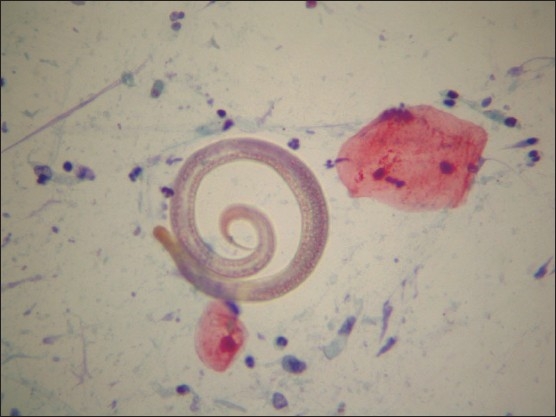
Cytologic examination of the bronchial wash showing a *S stercoralis* worm in a background containing some cellular debris (Papanicolaou stain ×400).

**Figure 2:**
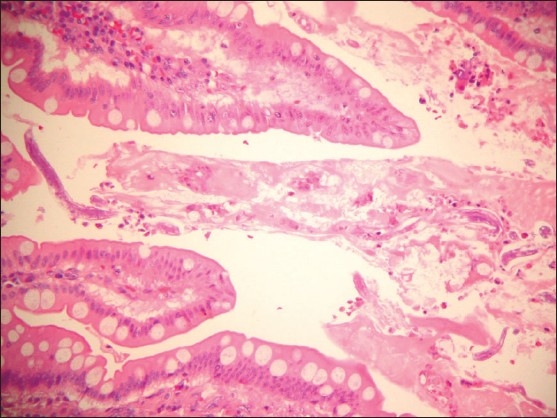
Low power view of the resected small bowel showing numerous larvae of *S stercoralis* in the mucinous material in between the villi (hematoxylin and eosin ×100).

**Figure 3:**
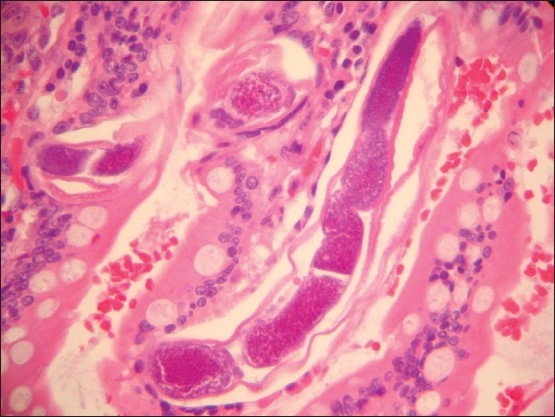
High power microscopic view showing larvae embedded within the small bowel villi. Notice the dark purplish organs surrounded by translucent sheath (coat) (hematoxylin and eosin ×400).

A 44-year-old Bangladeshi male was admitted to Hamad General Hospital on 31 December 2004 with progressive shortness of breath, fever, and productive cough with yellowish sputum of four days duration. His past history was remarkable for chronic obstructive pulmonary disease (COPD), secondary polycythemia, pulmonary hypertension, and old pulmonary tuberculosis. He was maintained on salbutamol inhaler, fluticasone, and salmetrol discus inhaler, theophylline and home oxygen therapy. He denied receiving systemic corticosteroids in the preceding year. He was an ex-smoker and alcohol drinker. Physical examination on admission revealed an ill looking, dyspneic patient with a temperature 38.4°C, blood pressure 135/90 mm Hg, pulse rate 160/minute, and respiratory rate 36/minute. Chest examination revealed expiratory wheezes and bilateral basal crepitations. Otherwise, physical examination was unremarkable. Laboratory investigations revealed hemoglobin 13 mg/dL, white blood cell count (WBC) 14.6×10^9^ /L (neutrophils 82%, lymphocytes 10%), platelets 25×10^9^ /L, glucose 11.4 mmol/L, creatinine 135 mmol/L, sodium 129 mmol/L, potassium 3.8 mmol/L. Arterial blood gases revealed pH 7.19, PCO_2_ 74.8 mm Hg, PO_2_ 96 mm Hg. Chest radiograph showed no lung infiltrates. Sputum culture grew *Pseudomonas aeruginosa*, sensitive to cefipime and ciprofloxacin; however, it was thought to represent colonization. A diagnosis of COPD with acute exacerbation was made, for which he was given intravenous ceftriaxone 2 gm once daily, azithromycin 500 mg once daily, methylprednisolone 40 mg three times daily, and salbutamol and ipratropium bromide nebulizers. He made a gradual improvement, and after seven days he was shifted to oral prednisolone 40 mg once daily and oral amoxicillin/clavulanic acid to complete two weeks of antibiotics. Prednisolone was given for a total of 13 days. Three weeks after admission he developed hemoptysis, chest tightness, and worsening cough. Sputum culture grew *P aeruginosa* for which he was given intravenous cefepime and ciprofloxacin. Sputum smear for acid fast bacilli (AFB) was negative. Because of worsening respiratory status and a chest radiograph showing an infiltrate involving the whole left lung and part of the right lung, bronchoscopy was performed on 27 January 2005. No endobronchial lesion was found. Antituberculous drugs in the form of isoniazid, rifampin, pyrazinamide, and ethambutol were started. Bronchioalveolar lavage and bronchial wash were negative for AFB but bacterial culture grew *P aeruginosa, Escherichia coli, and Klebisella pneumoniae*. Cytologic examination of the bronchial wash and lavage revealed bronchial epithelial cells, few squamous cells in the background of numerous *S stercoralis* worms (Figure 1) and bacteria with inflammatory cells and blood. Transbronchial biopsy showed only intraalveolar hemorrhage with anthracotic pigments, but was negative for granuloma, viral inclusions, or malignancy. He was treated with oral ivermectin 12mg once daily. Two days later he complained of vomiting, lower abdominal pain, abdominal distention, and constipation. Physical examination revealed abdominal distention, lower abdominal tenderness, and sluggish bowel sounds. A plain radiograph of the abdomen revealed distended bowel loops but no air fluid levels. Lapratomy was done. The findings at surgery were a dilated jejunum and ileum filled with stool. Microscopic examination of the resected part of the ileum bowel revealed acute inflammation, mucosal hemorrhage, and focal ulceration with abundant *S stercoralis* larvae (Figures 2 and 3). Postoperatively, he was mechanically ventilated. His postoperative course was complicated by hypotension and multiorgan failure and he expired after two days.

## CASE 2

A 39-year-old Nepali male was diagnosed with multiple myeloma complicated by spinal cord compression on 17 June 2007, for which he received radiotherapy and dexamethasone which was given for a total of six weeks. On 4 July 2007, he developed shortness of breath and hemoptysis associated with purpuric skin rash so he was transferred to intensive care unit. His past history was unremarkable. Physical examination revealed temperature 38°C, blood pressure 90/60 mm Hg, pulse rate 120/minute and respiratory rate 22/minute. Chest examination revealed bilateral fine crepitations up to the middle zone. Examination of the skin revealed palpable purpuric skin rash with vesicles involving the trunk and both upper thighs.

Laboratory investigations revealed hemoglobin 8.3 mg/dl, WBC 7.3×10^9^ /L (eosinophils 4.2%, lymphocyte 2.2%, neutrophil 92.1%), platelets 68×10^9^ /L, ESR creatine and electrolyte levels 81 mm/hr, ALT 109 U/L, AST 59 U/L, albumin 24 g/L, total protein 45 g/L. He had normal renal function tests. Arterial blood gases revealed pH 7.463, PCO_2_ 32 mm Hg, PO_2_ 63.6 mm Hg, and oxygen saturation of 93.3% on room air. Chest radiography showed diffuse bilateral reticular nodular and patchy infiltrate with multiple osteolytic lesions involving the lateral aspect of the right ribs (6^th^, 7^th^, and 8^th^ ribs). Sputum examination revealed larva of *S stercoralis* and culture grew *K pneumoniae*. Blood and urine cultures were negative. His condition deteriorated rapidly, so he was mechanically ventilated. He was treated with intravenous pipercillin-tazobactm 4.5 gm 8 hourly, ciprofloxacin 400 mg 12 hourly. A diagnosis of strongyloides hyperinfection syndrome was made, for which he was given intravenous albendazole 400 mg once daily for two days then oral ivermectin 9 mg once daily. Three stool samples for *Strongyloide* s were negative. Repeated sputum examination for *S stercoralis* was positive until 4 August after which ivermectin was discontinued. Skin biopsy showed evidence of herpes zoster with leukocytoclastic vasculitis with no evidence of cutaneous strongyloidiasis. Acyclovir 700 mg 8 hourly was added to his treatment regimen after receiving the biopsy result. Several days later he developed acute generalized motor neuropathy for which he underwent tracheostomy anticipating the long stay on ventilator. The cause of neuropathy was thought to be critical illness neuropathy.

On 5 August 2007, he developed fever, and neck stiffness. Lumber puncture revealed xanthochromic fluid. Cerebrospinal fluid (CSF) examination revealed WBC 130 cells/mL (neutrophils 25%, lymphocytes 80%), protein 3.57 mg/L, and glucose 4 mmol/L. The CSF was negative for India ink, bacterial latex antigen, AFB, or *S stercoralis* larvae with negative bacterial culture. He was started empirically on intravenous ampicillin 2 gm every 4 hours, cefepime 2 gm every 8 hours for possible meningitis, and ivermectin was restarted. Later on blood and CSF cultures came to be negative; however, urine and endotracheal cultures grew *P aeruginosa*. One day after starting antibiotics, the fever subsided and the patient became conscious. On 15 August the patient was found unconscious with fixed dilated pupils. CT head revealed extensive intraventricular hemorrhage with slight dilatation of all the ventricles and he expired one day later. The cause of intracerebral hemorrhage could not be ascertained since he had normal platelets, prothrombin, and activated partial thrombin times at the time of neurologic deterioration.

## DISCUSSION

Infection with *S stercoralis* was first reported in the year 1876 in French soldiers on duty in Vietnam.^2^ The first report of disseminated infection or hyperinfection dates back to 1966, when Cruz et al and Rogers et al. independently documented the occurrence of fatal strongyloidiasis with immunosuppression.^3^,^6^ The term hyperinfection is often used to denote autoinfection, a phenomenon in which the number of worms increases tremendously and worms are detectable in extraintestinal sites, especially the lung.^2^ Patients with impaired cellular immunity, especially those receiving long-term steroids, HTLV-1 and HIV-infected patients, and those with hematologic malignancies, transplant recipients, are at risk of developing the hyperinfection syndrome.^2^–^4^ Most of the patients who develop hyperinfection syndrome are receiving corticosteroids often for COPD. Pulmonary strongyloidiasis may mimic COPD exacerbation.^2^,^7^,^8^ Hyperinfection may develop as early as 20 days after the onset of corticosteroid therapy^9^ and as late as several years.^10^

The manifestations are a combination of gastrointestinal, pulmonary, and constitutional symptoms. The intestinal manifestations include severe cramping, abdominal pain, watery diarrhea, weight loss, nausea, vomiting, and occasionally gastrointestinal bleeding and small bowel obstruction.^4^ Pulmonary manifestations include mainly asthma-like symptoms such as cough and wheezing, and others such as pneumonia, pulmonary hemorrhage, pleural effusion, and acute respiratory failure.^4^,^5^ Hyperinfection may be complicated by bacterial infections and bacteremia caused by gut flora.^4^,^11^ Definitive diagnosis of strongyloidiasis is usually made on the basis of detection of larvae in stool or sputum.^2^,^12^ However, relying on stool studies alone for screening is inadequate. A single stool exam is said to be approximately 50% sensitive for making the diagnosis.^2^ The blood agar plate culture method, in which the serpiginous tracks of bacterial growth along the paths of motile larvae become apparent after 1 or 2 days of incubation at room temperature, is a preferred method because of its high sensitivity and ease of implementation in standard microbiology laboratories.^2^,^13^ Methods to sample duodenal fluid are more invasive and therefore less desirable. Serologic testing is now widely available and is sensitive but not specific.^2^ A single test does not reliably distinguish past from current infection.^2^,^14^ Albendazole, mebendazole, thiabendazole, and ivermectin have been shown to be effective for *S stercoralis* infection.^15^,^16^ Recently, there has been a change in the treatment of strongyloidiasis with more studies showing ivermectin as the drug of choice.^17^ Monitoring the response to treatment could be very difficult with detection of strongyloid larvae in the stool specimen because of the inconsistent shedding of the larvae.^18^

*S stercoralis* hyperinfection is rare in our area. Medline search from 1966 revealed only one report from Kuwait.^19^ In addition to the current two patients, we are aware of only one more case that was reported from Qatar in 1992.^20^ All three patients were from the Indian subcontinent, suggesting that *S stercoralis* hyperinfection in Qatar is a disease of the expatriate population especially those originating from the Indian Subcontinent.

The presentation in our patients was similar in many aspects to that described by others. Both patients presented initially with severe pulmonary disease that necessitated mechanical ventilation. These clinical features are nonspecific, therefore a high index of suspicion is needed for early diagnosis. Although, the mortality in patients with *S stercoralis* hyperinfection has been reported to be 50%, the fatal outcome in both of our patients is of concern and indicates the need for increasing awareness among physicians of this curable but potentially fatal disease. It is of interest that the first patient developed the hyperinfection syndrome after receiving a short course of corticosteroid therapy (13 days only). Both the fatal outcome in our patients and the development of the condition after a short course of corticosteroid therapy suggests the need to be vigilant in such patients to allow for early diagnosis and institution of appropriate treatment in order to avoid such unfortunate outcome. *S stercoralis* can remain quiescent in the host's intestine for as long as 30 years,^5^,^21^ becoming apparent when the host immunity decreases as a result of immunosuppressive drugs and/or disease. Physicians in the Gulf area should be aware of this. Although it is hard to draw conclusions from two cases, it may not be unreasonable to screen immunocompromised patients from endemic areas by serology before initiating steroid therapy to prevent the development of *Strongyloides* hyperinfection. Screening is not recommended for patients before starting short courses of corticosteroids for bronchial asthma or COPD, but may be considered in severe cases of bronchial asthma or COPD requiring recurrent and frequent steroids courses.

In conclusion, *S stercoralis* hyperinfection syndrome is a disease of immunocompromized patients, especially those who are receiving systemic steroids. Early diagnosis is a real challenge. Clinicians should be aware of the possibility of hyperinfection in immunocompromized patients, and that it may mimic other diseases leading to misdiagnosis.
